# Daily Thermal Fluctuations Experienced by Pupae via Rhythmic Nursing Behavior Increase Numbers of Mushroom Body Microglomeruli in the Adult Ant Brain

**DOI:** 10.3389/fnbeh.2016.00073

**Published:** 2016-04-19

**Authors:** Agustina Falibene, Flavio Roces, Wolfgang Rössler, Claudia Groh

**Affiliations:** Department of Behavioral Physiology and Sociobiology, Biozentrum, University of WürzburgWürzburg, Germany

**Keywords:** *Camponotus* ants, brood translocation, temperature, olfaction, vision, synaptic plasticity, mushroom body, microglomeruli

## Abstract

Social insects control brood development by using different thermoregulatory strategies. *Camponotus mus* ants expose their brood to daily temperature fluctuations by translocating them inside the nest following a circadian rhythm of thermal preferences. At the middle of the photophase brood is moved to locations at 30.8°C; 8 h later, during the night, the brood is transferred back to locations at 27.5°C. We investigated whether daily thermal fluctuations experienced by developing pupae affect the neuroarchitecture in the adult brain, in particular in sensory input regions of the mushroom bodies (MB calyces). The complexity of synaptic microcircuits was estimated by quantifying MB-calyx volumes together with densities of presynaptic boutons of microglomeruli (MG) in the olfactory lip and visual collar regions. We compared young adult workers that were reared either under controlled daily thermal fluctuations of different amplitudes, or at different constant temperatures. Thermal regimes significantly affected the large (non-dense) olfactory lip region of the adult MB calyx, while changes in the dense lip and the visual collar were less evident. Thermal fluctuations mimicking the amplitudes of natural temperature fluctuations via circadian rhythmic translocation of pupae by nurses (amplitude 3.3°C) lead to higher numbers of MG in the MB calyces compared to those in pupae reared at smaller or larger thermal amplitudes (0.0, 1.5, 9.6°C), or at constant temperatures (25.4, 35.0°C). We conclude that rhythmic control of brood temperature by nursing ants optimizes brain development by increasing MG densities and numbers in specific brain areas. Resulting differences in synaptic microcircuits are expected to affect sensory processing and learning abilities in adult ants, and may also promote interindividual behavioral variability within colonies.

## Introduction

Temperature has a major impact on the development and life history of insects. The thermal range preferred during development is species-specific, and substantial deviations from its boundaries can cause brood abnormalities, increase mortality, or influence developmental times. Within these boundaries, some insects show optimal development when reared under constant temperature regimes and others when exposed to fluctuating temperatures, even when these regimes are defined by the same mean temperature (reviewed in Ratte, 1985). Temperature not only affects quantitative aspects of insect development, i.e., growth parameters, but also qualitative (differentiation) aspects, including changes in behavioral traits (e.g., Tautz et al., 2003; Jones et al., 2005; Becher et al., 2009; Weidenmüller et al., 2009).

Most social insects (ants, termites, social bees, and wasps) are, to some extent, able to regulate the temperature their brood is exposed to by using different strategies and mechanisms (reviewed by Seeley and Heinrich, 1981; Jones and Oldroyd, 2006). Honeybees, for example, maintain a constant brood temperature around 34.8°C (Himmer, 1927), which is controlled by generating metabolic heat through flight muscle activity, or by cooling via fanning and water evaporation (Seeley and Heinrich, 1981; Heinrich, 1993). In ants, only a few species rely on metabolic heat production for thermoregulation (*Eciton* bivouacking army ants: Franks, 1989; *Formica* wood ants: Rosengren et al., 1987), while most ant species require external heat sources to elevate their body temperature. However, these ant species regulate the brood temperature by other mechanisms such as nest site selection, nest architecture, and colony migration among others (Seeley and Heinrich, 1981; Jones and Oldroyd, 2006).

In some ant species, avoidance of extreme temperatures and a fine control of brood rearing conditions is achieved by moving the immobile brood inside the nest (e.g., *Camponotus*: Roces and Núñez, 1989; *Solenopsis*: Porter and Tschinkel, 1993; Pranschke and Hooper-Bùi, 2003; Penick and Tschinkel, 2008; *Acromyrmex*: Bollazzi and Roces, 2002). In the nectar-feeding ant *Camponotus mus*, nurse workers show a particularly interesting behavior. They translocate the immobile brood twice a day to different preferred temperatures following a circadian rhythm at times that correlate with the daily temperature extremes (Roces and Núñez, 1989, 1995, 1996; Roces, 1995). When workers and brood are placed on an artificial nest offering a thermal gradient, nurses move the brood from 27.5 to 30.8°C at the middle of the photophase (2 p.m.), and 8 h later (10 p.m.) the brood is moved back to 27.5°C (Video in Supplementary Material). Nurse workers actively seek to expose the brood to these thermal fluctuations of ~3.3°C amplitude, and the translocation behavior stops when this thermal regime is artificially simulated (Roces and Núñez, 1989, 1996). Thermoregulatory behavior represents a large energy investment by nurse workers. During the translocation events, pupae are moved first, larvae in the second place, and finally eggs are translocated. Under starvation, when protein necessary for larval growth is not available, only pupae and last instar larvae (i.e., in which pupation has already been triggered) are translocated (Roces and Núñez, 1989). This indicates that fluctuating temperature regimes as actively provided by nurse ants to the brood are of particular relevance for postembryonic development during the pupal phase. However, their specific effects remain elusive, and differential effects of fluctuating and constant temperatures are not evident in growth parameters such as developmental time or mortality in *C. mus* (Roces and Núñez, 1989).

**Figure 1:**
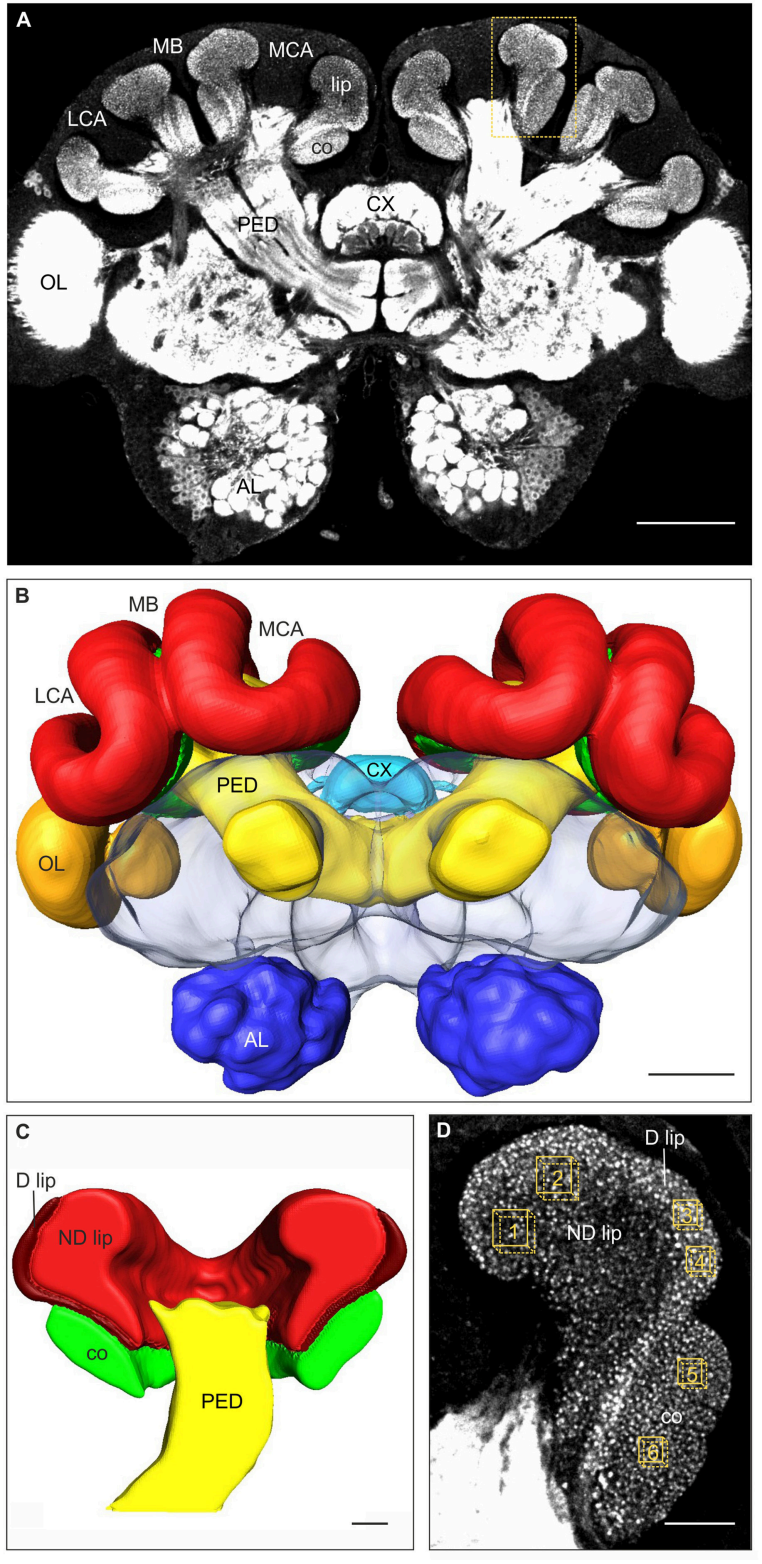
**Immunofluorescence staining with anti-synapsin antibody, 3D reconstruction, and MG quantification in brains of 2-day old *C. mus* workers. (A)** Frontal overview of a brain in a central plane. Higher magnification of the area marked by the box is shown in **(D)**. **(B)** 3D reconstruction of the brain showing the major neuropils. **(C)** Frontal plane of the 3D reconstruction of the MB calyx showing the visual collar, and the dense (D) and non-dense (ND) olfactory lip subregions. **(D)** Higher magnification of the medial MB calyx (box shown in **A**). Boxes 1–2, 3–4, and 5–6 indicate the volumes used for quantification of the presynaptic boutons in the ND lip, D lip and collar regions of the MB calyces, respectively. AL, antennal lobe; co, collar; CX, central complex; LCA, lateral calyx; MB, mushroom bodies; MCA, medial calyx; OL, optic lobe; PED, pedunculus. Scale bars: **(A,B)** 100 μm; **(C,D)** 20 μm.

During the pupal phase in holometabolous insects such as Hymenoptera, major anatomical changes take place. The central nervous system is extensively remodeled, including for example the metamorphic reorganization of the mushroom bodies (MBs) (ants: Ishii et al., 2005; bees: Farris et al., 1999; Schröter and Malun, 2000; Fahrbach, 2006; Groh and Rössler, 2008). The MBs are high-level sensory integration centers in the insect brain involved in learning, memory formation, and orientation (Erber et al., 1980; Menzel, 1990, 1999; Hammer and Menzel, 1995; Strausfeld et al., 1998; Heisenberg, 2003; Davis, 2005; Giurfa, 2007; Hourcade et al., 2010; Falibene et al., 2015). In social Hymenoptera, the MBs are particularly large and receive olfactory (MB calyx lip) and visual (MB calyx collar) information (Gronenberg, 2001; Groh et al., 2014; Yilmaz et al., 2016; Figures 1A–C). In the calyces, projection neurons (PNs) originating from the antennal and optic lobes terminate in large presynaptic boutons surrounded by postsynaptic dendritic spines of MB intrinsic neurons (Kenyon cells, KCs) forming characteristic synaptic complexes termed microglomeruli (MG) (Ganeshina and Menzel, 2001; Gronenberg, 2001; Frambach et al., 2004; Groh et al., 2004, 2006; Seid and Wehner, 2008). Several studies in ants and bees have demonstrated a high level of plasticity of MG associated with behavioral transitions, age, sensory exposure, as well as associative learning and long-term memory formation (Seid et al., 2005; Seid and Wehner, 2009; Hourcade et al., 2010; Stieb et al., 2010, 2012; Groh et al., 2012; Scholl et al., 2014; Falibene et al., 2015; Muenz et al., 2015; Yilmaz et al., 2016). In the honeybee, differences in constant temperatures experienced during pupal development were shown to affect the MB-calyx neuroarchitecture in the adult brain (Groh et al., 2004, 2006).

In this study we investigated whether daily thermal fluctuations experienced by developing *C. mus* pupae during circadian rhythmic nursing behavior affect the neuroarchitecture of the adult brain. We compared the effects of daily thermal fluctuations of different amplitudes (with the same mean temperature), and different constant temperatures. We focused our study on the MBs as they are involved in high-level sensory integration, learning and memory formation, and might underlie variability in behavioral traits. Using immunolabeling of large presynaptic boutons in complete brains (whole-mounts), we compared neuropil volumes together with synaptic bouton densities in olfactory and visual MB regions of young adult ants reared at different thermal regimes.

## Materials and methods

### Animals

Experiments were conducted using six different colonies of *C. mus*. Each colony was founded by a single queen (collected by O. Geissler in La Coronilla, Uruguay in 2008 and 2009), comprised ~100–250 individuals, and was maintained in the laboratory under controlled conditions (25°C, 50% relative humidity, 12:12 h light:dark cycles). Twenty four hours before starting the experiments, all pupae were removed from the nests in order to assure that all new pupae found at the beginning of the experiment had spun the cocoon not earlier than 24 h before. All newly spun cocoons were collected every morning at the same time and placed in groups of up to three in small plastic boxes (9 × 9 × 6 cm^3^) with wet plaster floors. Five to six adult workers were paint-labeled (Edding 751 paint marker, Edding International GmbH, Ahrensburg, Germany) on the thorax, and placed together with the pupae to provide them care. All individuals (pupae and workers) in the same box belonged to the same colony. Boxes were then placed in high-precision incubators (Memmert HPP110, Schwabach, Germany; Panasonic MIR-154-PE, Bad Nenndorf, Germany), and maintained in constant darkness under one thermal treatment, as detailed below.

Boxes in the incubators were checked for individual survival and newly eclosed workers every morning at the same time. Developmental time (days from cocoon spinning to adult eclosion) was computed for each individual. After eclosion, workers were kept for another 48 h under the same temperature regime, similar to natural conditions where young ants remain close to the developing brood during the first days of their adult life and to continuously experience the brood temperature regimes. Ants were then collected for brain dissection and synaptic labeling (see below). Mortality was computed when imaginal molting did not occur (pre-eclosion mortality) or when the emerged ant died during the 48 h time window until dissection (post-eclosion mortality). Thorax lengths (mm, distance from the anterior edge of the pronotum to the petiole) were measured in all dissected ants and used as a measure of body size. All procedures were performed in accordance with local and national guidelines for the use of animals in research.

### Thermal treatments

#### Fluctuating temperatures

Groups of pupae were reared under daily fluctuating temperatures with different thermal amplitudes, but the same mean temperature, as detailed in Table 1. Thermal regimes were set based on the daily thermal oscillation experienced by pupae via the nurses' thermal preferences for brood translocation, i.e., at 2 p.m. pupae experienced a change from lower to higher temperatures, and 8 h later (10 p.m.) they were moved back to lower temperatures, where they remained for 16 h (Roces and Núñez, 1989). We compared the effects of the daily thermal program naturally preferred by nurses (amplitude 3.3°C; 16 h at 27.5°C–8 h at 30.8°C; mean temperature 28.6°C) with those of lower (amplitude 1.5°C; 28.1–29.6°C) and higher (amplitude 9.6°C; 25.4–35.0°C) thermal fluctuations with the same mean temperature. In the latter one, the maximal temperature of the range coincides with the maximal tolerated temperature by nurses during brood care (Roces and Núñez, 1995). Finally, we also investigated a group reared at a constant temperature of 28.6°C (amplitude 0.0°C), which corresponds to the mean temperature experienced via the nurses' thermal preferences (Roces and Núñez, 1989).

**Table 1:** **Daily fluctuating temperature with different thermal amplitude regimes experienced by the ants during the pupal phase**.

**Amplitude (°C)**	**Daily temperature (°C)**		
	**10 p.m.–2 p.m. (16 h)**	**2 p.m.–10 p.m. (8 h)**	**Mean**
0.0	28.6	28.6	28.6
1.5	28.1	29.6	28.6
3.3	27.5	30.8	28.6
9.6	25.4	35.0	28.6

*The gray bar indicates the daily thermal program preferred by C. mus nurses to maintain the brood*.

#### Constant temperature

In an independent experiment, pupae were reared under two different constant temperatures: 25.4 or 35.0°C. These represented the two extreme temperatures experienced by the pupae in the larger thermal amplitude regime in the fluctuating temperature experiment.

In both experimental series, relative humidity in the incubators was maintained within the range of 60–80%. In the fluctuating temperature regimes, changes between the two temperatures of the ranges took no more than 40 min, which approximates the time needed by small groups of nurses in colonies of up to 80 workers to translocate all the brood (Roces and Núñez, 1989).

### Immunostaining of presynaptic terminals

To analyze the neuroarchitecture of the MB calyces, we used a well-established protocol for immunolabeling of presynaptic terminals in whole-mount preparations (Groh et al., 2012, 2014; Falibene et al., 2015; Muenz et al., 2015). By using anti-synapsin antibodies, the central presynaptic bouton of each synaptic complex (MG) in the MB calyces can be easily identified. Preliminary experiments showed that in 1-day old ants, synapsin immunoreactivity was not exclusively localized in the PN axonal boutons, but still distributed along its axons. Therefore, workers were processed 48 h after eclosion. In brief, ants were anesthetized on ice, their brains dissected in ant Ringer solution and fixed in 4% formaldehyde in phosphate-buffered saline (PBS; pH 7.2) overnight. Brains were then washed in PBS, permeabilized by Triton X-100 in PBS (2%, 1 × 10 min and 0.1%, 2 × 10 min) and blocked with 2% normal goat serum (NGS, 1 h; Jackson ImmunoResearch Laboratories, PA, USA). Preparations were then incubated in the primary mouse monoclonal antibody to the *Drosophila* synaptic-vesicle-associated protein synapsin I (1:50, 3 days; SYNORF1, kindly provided by Dr. E. Buchner, University of Würzburg, Germany). Afterwards, brains were washed in PBS and incubated in CF488A goat anti-mouse secondary antibody (1:250, 2 days; Biotium, CA, USA). Brains were then fixed again overnight and subsequently washed in PBS, dehydrated through an ascending ethanol series and mounted on aluminum slides in methyl salicylate (M-2047, Sigma Aldrich, Steinheim, Germany).

### Laser-scanning confocal microscopy

Whole-mount preparations were visualized by using a laser-scanning confocal microscope (Leica TCS SP2, Leica Microsystems, Wetzlar, Germany) equipped with an argon/krypton laser. The brain overview was done by using a 20x 0.7 NA imm objective with a 1x digital zoom. For its 3D reconstruction, 4 μm optical sections through the entire depth of the brain were performed. For volume measurements of MB calyx subregions, optical sections were taken at 3 μm intervals through the entire left medial calyx using a 20x 0.7 NA imm objective and a 3x digital zoom. For quantification of synapsin-immunoreactive (IR) boutons, optical sections of the innermost part of the right medial calyx were scanned at 0.5 μm intervals through a depth of 10 μm using a 63x 1.4 NA imm objective and a 3x digital zoom. All optical sections were taken at a resolution of 1024 × 1024 pixels.

### Data acquisition

Images were processed using the 3D reconstruction software AMIRA v. 5.3 (FEI Visualization Sciences Group, Düsseldorf, Germany) following well-established procedures (Groh et al., 2012, 2014; Falibene et al., 2015; Muenz et al., 2015). In order to reconstruct the entire brain and to measure the lip and collar neuropil volumes, the outer border of each region on each section were traced and 3D reconstructed. We distinguished between the non-dense (ND) and dense (D) lip subregions, first described in *Atta* leaf-cutting ants (Groh et al., 2014) and also found in *Acromyrmex* (Falibene et al., 2015) and *Camponotus* ants (Yilmaz et al., 2016). We estimated the relative ND and D lip proportions by measuring their volumes separately in 12 brains (3 brains per fluctuating temperatures treatment), where differential staining between the two subregions was very clear at lower magnification (Figures 1C,D). Using this relative proportion, we then estimated the volume of the ND and D lip from the total lip volume in individual brains. Bouton densities in the ND lip, D lip and collar, respectively, were quantified by counting the synapsin-IR boutons in defined volumes (ND lip: 1000 μm^3^, 129 × 129 voxel; D lip and collar: 490 μm^3^, 90 × 90 voxel, Figure 1D). Two samples each were taken and averaged per calyx region and brain. Bouton density was calculated as number of boutons per μm^3^. The total number of synapsin-IR boutons per calyx in each region was then calculated by multiplying the density (boutons/μm^3^) by the corresponding neuropil volumes (μm^3^).

### Statistical analyses

Analyses were carried out using InfoStat (v. 2012, Di Renzo et al., 2012) and IBM SPSS (v. 22) statistical software. Developmental time was compared by using Kruskal–Wallis test followed by Dunn comparisons when necessary. *G*-tests were applied for comparing pupal mortality among treatments. Volumetric variables and bouton densities were compared by using Analysis of Covariance (ANCOVA) with individual thorax length (referred as “size”) included as a covariate. Normality and homogeneity of variance assumptions were tested in all cases. Statistically significant ANCOVA tests were followed by Tukey test comparisons. In those cases in which the covariate thorax length was significant, relationships between variables were analyzed by Spearman rank-correlation coefficients. Spearman rank-correlation coefficients tests were used also to analyze the relationship between developmental time, ant size, and thermal experience. The significance level used was 5% in all cases.

## Results

### Pupal developmental time and mortality rate

**Figure 2:**
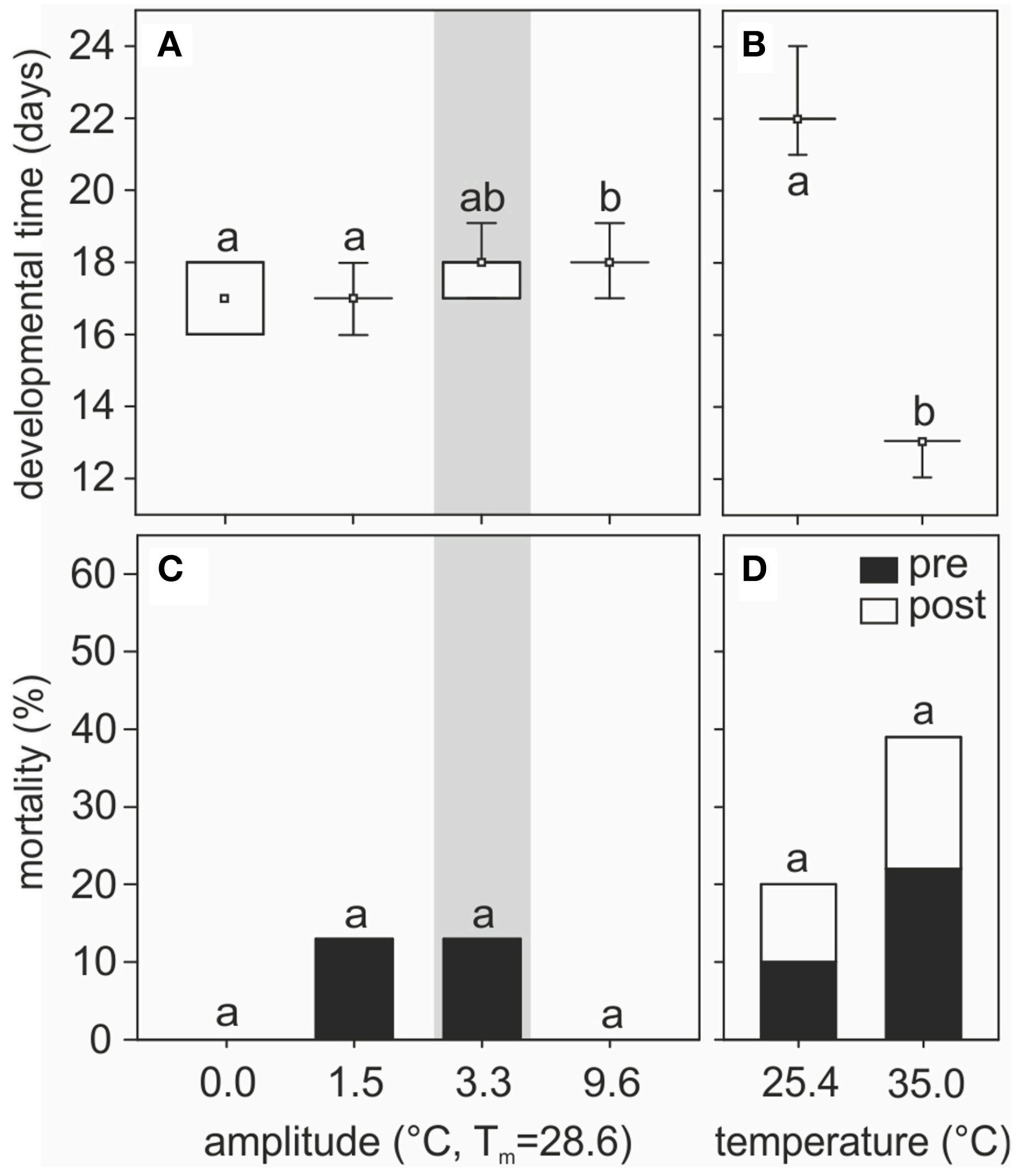
**(A,B)** Pupal developmental time and **(C,D)** mortality rates of individuals reared at **(A,C)** different amplitudes of fluctuating temperature or **(B,D)** constant temperature regimes. Developmental time is shown as boxplots: boxes show quartiles, whiskers provide the minimum and maximum values, and small squares represent medians. Percentage of pre- and post- eclosion mortality for each group is indicated by black and white bars, respectively. Gray area indicates the temperature regime selected by nurses for brood translocation. Different letters show significant differences among different amplitudes of fluctuating temperature or between constant temperatures regimes. Developmental time: *N*_0_ = 15, *N*_1.5_ = 14, *N*_3.3_ = 13, *N*_9.6_ = 16, *N*_25.4_ = 18, *N*_35_ = 18. Mortality: *N*_0_ = 15, *N*_1.5_ = 16, *N*_3.3_ = 15, *N*_9.6_ = 16, *N*_25.4_ = 20, *N*_35_ = 23.

Temperature experienced during the pupal phase affected the time of pupal development until eclosion. Under temperature regimes fluctuating around the same mean yet with different amplitudes, pupal developmental time varied with the thermal amplitude (*H*_3, *N* = 58_ = 20.93, *p* = 0.0001, Kruskal–Wallis, Figure 2A). Pupae reared at 0.0 and 1.5°C amplitude regimes required shorter developmental times (between 16 and 18 days) than those reared under a 9.6°C amplitude of thermal fluctuation but the same mean temperature (between 17 and 19 days). Pupae reared at temperature fluctuations selected by nurses (amplitude 3.3°C) required between 17 and 19 days to complete their development and, on average, this group did not significantly differ from the others. Under constant temperatures, a large difference was evident between groups of pupae reared at 25.4 and 35.0°C (21–24 and 12–13 days, respectively, *H*_1, *N* = 36_ = 26.27, *p* < 0.0001, Kruskal–Wallis, Figure 2B).

Mortality did not significantly differ among groups of pupae reared at different fluctuating temperature regimes (*G* = 5.83, *p* = 0.12, *G*-test, Figure 2C) or at two different constant temperatures (*G* = 1.89, *p* = 0.17, *G*-test, Figure 2D). On average, however, mortality was higher at relatively lower (25.4°C: 20% mortality) and higher (35.0°C: 39% mortality) constant temperatures, compared to mortality at 28.6°C constant temperature (0%) or fluctuating temperatures (0–13%). Interestingly, with fluctuating temperature regimes all non-surviving pupae died before eclosion, whereas in constant temperature regimes ~50% of the non-surviving individuals died 1 or 2 days post-emergence.

Ants reared under different thermal treatments did not differ in thorax length (*fluctuating temperature*: *H*_3, *N* = 58_ = 6.33, *p* = 0.10; *constant temperature*: *H*_1, *N* = 31_ = 2.89, *p* = 0.09; Kruskal–Wallis, Table 2). However, under fluctuating temperature regimes with 9.6°C amplitude, developmental time positively correlated with the thorax length of the emerged workers, i.e., larger ants needed a longer time to develop (Table 2). No statistically significant correlation was found between developmental time and thorax length for the other fluctuating temperature treatments or under constant temperatures.

**Table 2:** **Correlation between developmental time and thorax length for fluctuating and constant temperature regimes**.

**Treatment**	**Thorax length (mm)**	***n***	**Spearman**	***p*-value**
**AMPLITUDE (**°**C**, ***T**_*m*_* = **28.6)**				
0.0	1.85 (1.66–1.99)	15	0.34	0.218
1.5	1.93 (1.63–2.30)	14	0.28	0.330
3.3	1.84 (1.61–2.13)	13	0.48	0.095
9.6	1.92 (1.72–2.12)	16	0.71	0.026^*^
**TEMPERATURE (**°**C)**				
25.4	1.97 (1.79–2.13)	16	0.26	0.341
35.0	1.91 (1.80–2.03)	15	0.00	0.999

*Mean values of thorax lengths are shown, minimum and maximum values of each group are indicated between parentheses. See Section Results for pupal developmental times. In each case, only ants that were still alive 48 h after eclosion (i.e., ants later dissected for brain immunolabeling) were considered. The light gray bar indicates the daily thermal program preferred by C. mus nurses to maintain the brood. Asterisks denote significant differences*.

### Mushroom body synaptic organization

#### Temperature effects on the MB calyx volume

**Figure 3:**
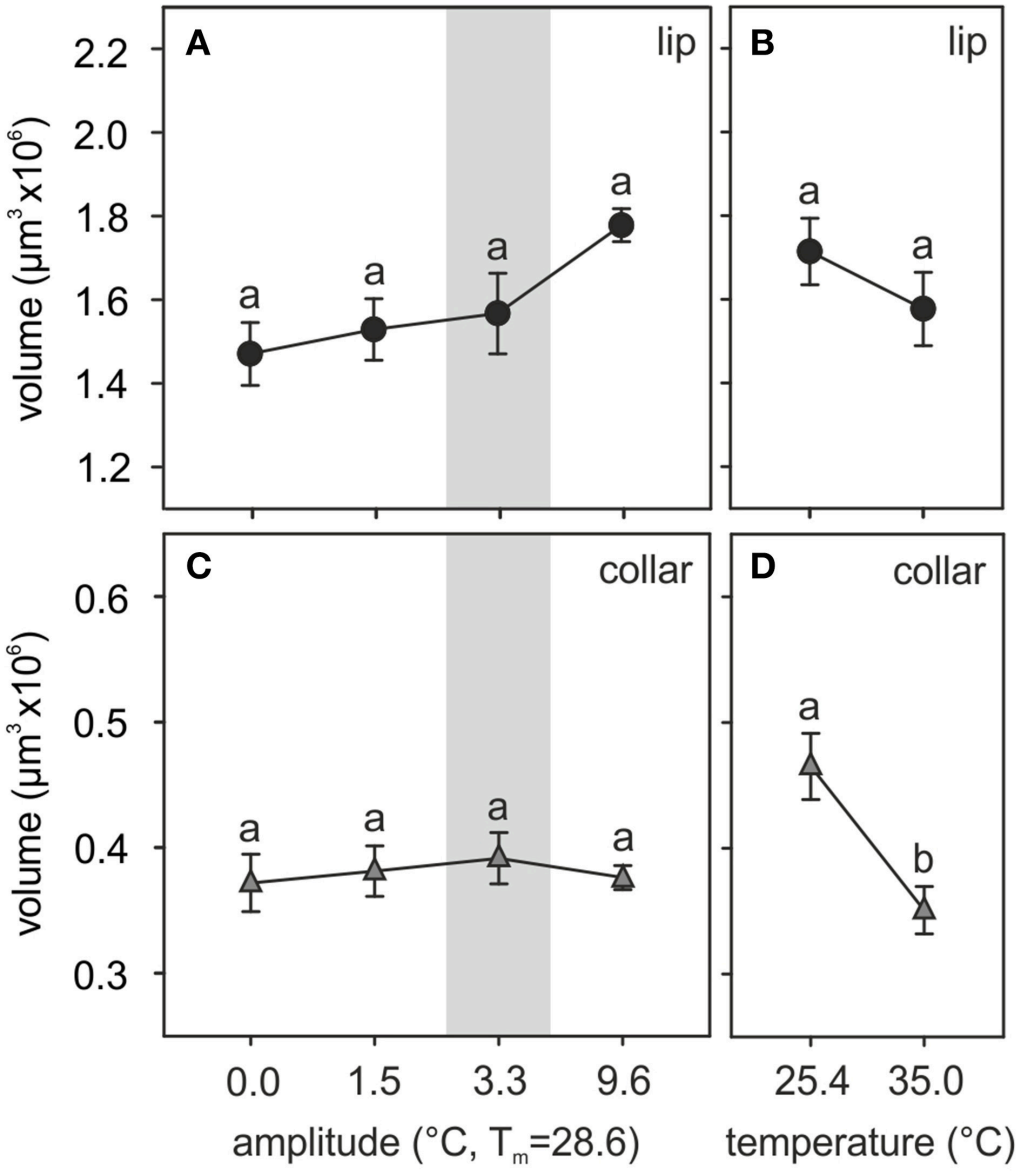
**Temperature effects on the adult MB calyx volume**. **(A,B)** Volume of the olfactory lip and **(C,D)** the visual collar input regions of the MB calyces of ants reared at **(A,C)** different amplitudes of fluctuating temperature but the same mean temperature or **(B,D)** different constant temperature regimes. Gray area shows the temperature regime selected by nurses for brood translocation. Symbols represent the mean value of each group and lines the S.E. Different letters indicate significant differences among treatments with different amplitudes or between those with different constant temperatures. Lip and collar: *N*_0_ = 13, *N*_1.5_ = 9, *N*_3.3_ = 12, *N*_9.6_ = 10, *N*_25.4_ = 14, *N*_35_ = 12.

An example of a 2-day old adult ant brain labeled with anti-synapsin and its 3D reconstruction is shown in Figure 1. The olfactory lip region of the MBs showed no statistically significant volumetric differences among ants that had experienced different fluctuating temperature treatments [total lip volume, *amplitude*: *F*_(3, 39)_ = 2.66, *p* = 0.06; *covariate size*: *F*_(1, 39)_ = 0.04, *p* = 0.85; ANCOVA; Figure 3A]. Those ants that experienced a high thermal amplitude (9.6°C) during the pupal phase tended to develop bigger lip regions, but differences were not statistically significant in comparison with those reared under smaller thermal amplitudes. No differences were found for the total lip volume between different constant temperatures [*temperature*: *F*_(1, 23)_ = 1.11, *p* = 0.30; *covariate size*: *F*_(1, 23)_ = 0.01, *p* = 0.93; ANCOVA; Figure 3B].

Two structurally different subregions were observed in the calyx lip based on the density of the synapsin staining patterns. The large inner part, the ND lip, showed an overall weaker staining and presynaptic boutons appeared more dispersed compared to those in the outer region, the D lip (Figures 1C,D). The ND lip in ants reared under 0.0, 1.5, 3.3, and 9.6°C amplitude comprised ~81, 84, 84, and 79% of the total lip volume, respectively. No significant differences were found in the relative volume proportion of these two regions among ants that had experienced different fluctuating temperatures (*H*_3, *N* = 12_ = 6.58, *p* = 0.08, Kruskal–Wallis).

Similarly, the visual collar showed no significant volumetric variations among ants reared at different fluctuating temperatures [*amplitude*: *F*_(3, 39)_ = 0.18, *p* = 0.91; *covariate size*: *F*_(1, 39)_ = 0.08, *p* = 0.78; ANCOVA; Figure 3C], but significant differences were found between groups of ants that had been reared at different constant temperatures [*temperature*: *F*_(1, 23)_ = 9.19, *p* = 0.006; *covariate size*: *F*_(1, 23)_ = 0.33, *p* = 0.57; ANCOVA; Figure 3D]. Relatively lower constant temperatures (25.4°C) during the pupal phase promoted the development of bigger visual collar regions (compared to 35.0°C) in adult ants.

In summary, the temperature experienced by the pupae had only little effects on the MB calyx volumes in the adult brain. However, neuropil volumes may not be the best indicators as neuronal processing capacities are determined by numbers of neurons and their interconnection complexity (Chittka and Niven, 2009; Groh et al., 2012, 2014). We therefore quantified the densities and extrapolated total numbers of synaptic complexes in the adult MB calyx subregions following various temperature treatments.

#### Temperature effects on MB calyx synaptic bouton densities

**Figure 4:**
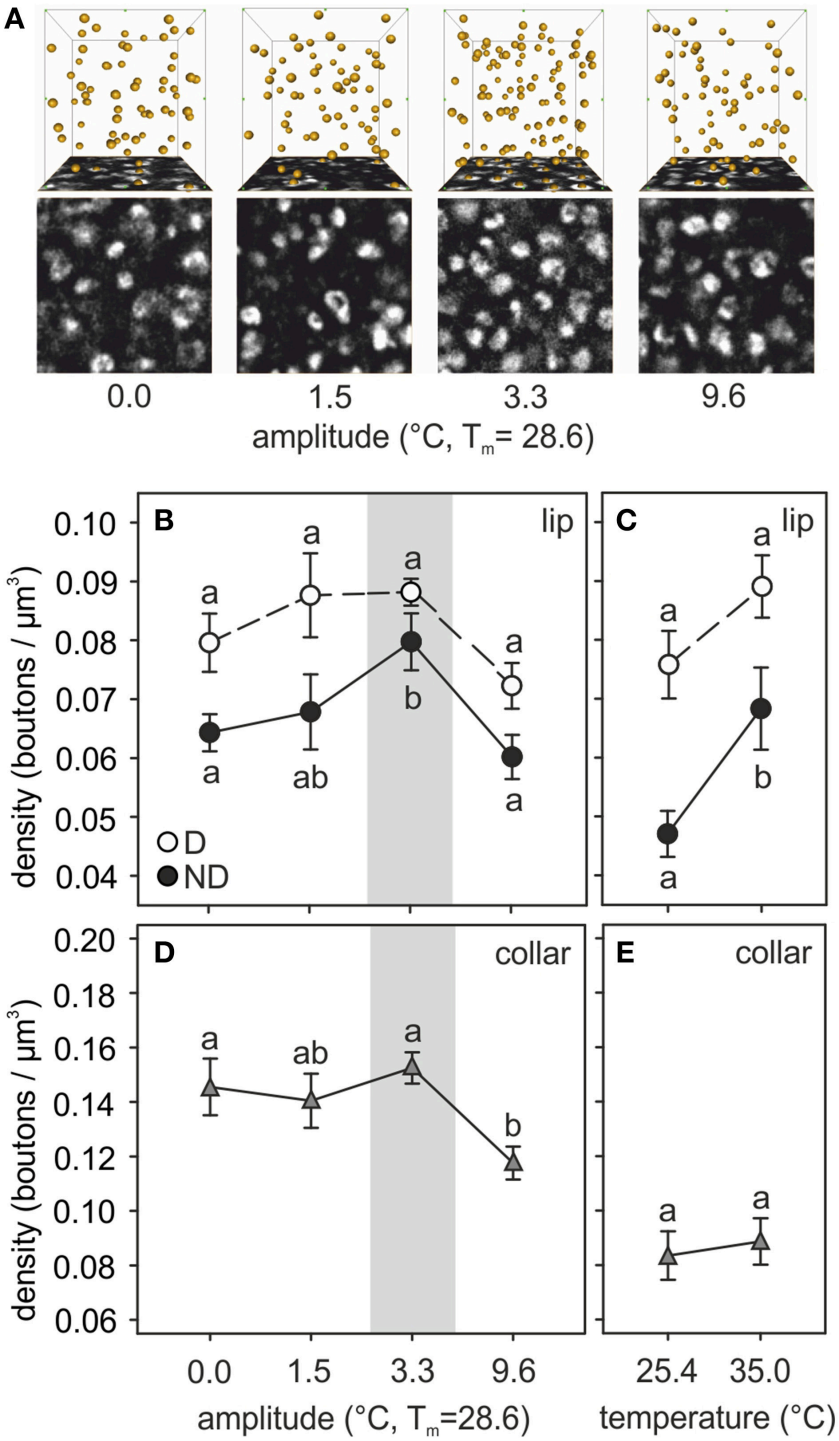
**Temperature effects on synaptic bouton densities. (A)** Examples of synapsin-IR bouton quantification in the ND lip of ants reared at 0.0, 1.5, 3.3, and 9.6°C of thermal amplitude (mean temperature, *T*_*m*_: 28.6°C). Single confocal image of a 10 × 10 μm^2^ synapsin-stained area in the ND lip (bottom) and 3D reconstruction (top) of the position of the boutons visualized by AMIRA in a 1000 μm^3^–volume (10 × 10 × 10 μm^3^). Each yellow sphere marks the center of a synapsin-IR bouton. **(B–E)** Presynaptic bouton densities in **(B,C)** the olfactory D and ND lip and **(D,E)** the visual collar input regions of the MB calyces of ants **(B,D)** reared at different amplitudes of fluctuating temperature but the same mean temperature or **(C,E)** different constant temperature regimes. Gray area shows the temperature regime selected by nurses for brood translocation. Symbols represent the mean value of each group and lines the S.E. Different letters indicate significant differences among amplitudes or between constant temperatures. Lip: *N*_0_ = 13, *N*_1.5_ = 9, *N*_3.3_ = 10, *N*_9.6_ = 11, *N*_25.4_ = 11, *N*_35_ = 11; collar: *N*_0_ = 10, *N*_1.5_ = 8, *N*_3.3_ = 11, *N*_9.6_ = 10, *N*_25.4_ = 11, *N*_35_ = 10.

Brains of ants reared at different amplitudes of fluctuating temperature significantly differed in the density of synapsin-IR boutons in the ND subregion of the olfactory lip of the MB calyces (Figure 4). ND lip boutons had the highest packing density in those ants reared at 3.3°C thermal amplitude [*amplitude*: *F*_(3, 38)_ = 6.31, *p* = 0.0014; *covariate size*: *F*_(1, 38)_ = 19.75, *p* = 0.0001; ANCOVA; Figures 4A,B], the regime that resembled the daily thermal preferences of nurses for brood rearing. Workers reared under a constant temperature of 28.6°C (amplitude 0.0°C) or under a high temperature variation (amplitude 9.6°C) showed significantly lower ND lip bouton densities than those under a thermal amplitude of 3.3°C. Workers reared under 1.5°C thermal amplitude showed intermediate values. Furthermore, bouton densities in the ND lip significantly correlated with ant size (Table 3). In the constant temperature series, the ND lip bouton density was significantly higher in ants reared at 35.0°C compared to those reared at 25.4°C [*temperature*: *F*_(1, 19)_ = 7.59, *p* = 0.013; *covariate size*: *F*_(1, 19)_ = 0.69, *p* = 0.42; ANCOVA; Figure 4C]. Most interestingly, variations in bouton densities was subregion-specific within the lip as no significant differences among fluctuating temperature treatments were found for the D lip subregion [*amplitude*: *F*_(3, 38)_ = 2.86, *p* = 0.05; *covariate size*: *F*_(1, 38)_ = 2.00, *p* = 0.17; ANCOVA; Figure 4B], nor between groups that had experienced constant temperatures [*temperature*: *F*_(1, 19)_ = 1.08, *p* = 0.31; *covariate size*: *F*_(1, 19)_ = 3.99, *p* = 0.06; ANCOVA; Figure 4C]. D lip bouton density was lower in ants reared under a 9.6°C amplitude, but differences were not statistically significant in comparison with those ants reared under smaller thermal amplitudes.

**Table 3:** **Correlation between synaptic bouton density in the non-dense (ND) MB calyx lip and thorax length for fluctuating and constant temperature regimes**.

**Treatment**	**ND lip density (b/μm^3^)**	***n***	**Spearman**	***p*-value**
**AMPLITUDE (**°**C**, ***T**_*m*_* = **28.6)**				
0.0	0.064 (0.049–0.077)	13	0.23	0.500
1.5	0.068 (0.042–0.096)	9	0.73	0.038^*^
3.3	0.081 (0.054–0.103)	10	0.85	0.003^*^
9.6	0.060 (0.040–0.076)	11	0.26	0.288
**TEMPERATURE (**°**C)**				
25.4	0.047 (0.025–0.067)	11	−0.26	0.444
35.0	0.068 (0.028–0.097)	11	0.82	0.002^*^

*Mean values of ND lip densities are shown, minimum and maximum values of each group are indicated between parentheses. See Table 2 for thorax lengths. The light gray bar indicates the daily thermal program preferred by C. mus nurses to maintain the brood. Asterisks denote significant differences*.

The temperature experienced during the pupal phase also affected the development of the visual MB collar. Statistical differences in bouton densities were found among ants that had experienced different fluctuating thermal regimes [*amplitude*: *F*_(3, 34)_ = 4.68, *p* = 0.007; *covariate size*: *F*_(1, 34)_ = 1.90, *p* = 0.18; ANCOVA; Figure 4D]. Ants reared under 0.0, 1.5, and 3.3°C thermal amplitudes did not differ in the collar bouton densities. However, ants that had experienced a high amplitude of 9.6°C showed significantly lower bouton densities. In the constant temperature series, no changes in bouton densities were found between treatments [*temperature*: *F*_(1, 18)_ = 0.08, *p* = 0.78; *covariate size*: *F*_(1, 18)_ = 0.08, *p* = 0.78; ANCOVA; Figure 4E].

#### Temperature effects on MB synaptic bouton numbers

**Figure 5:**
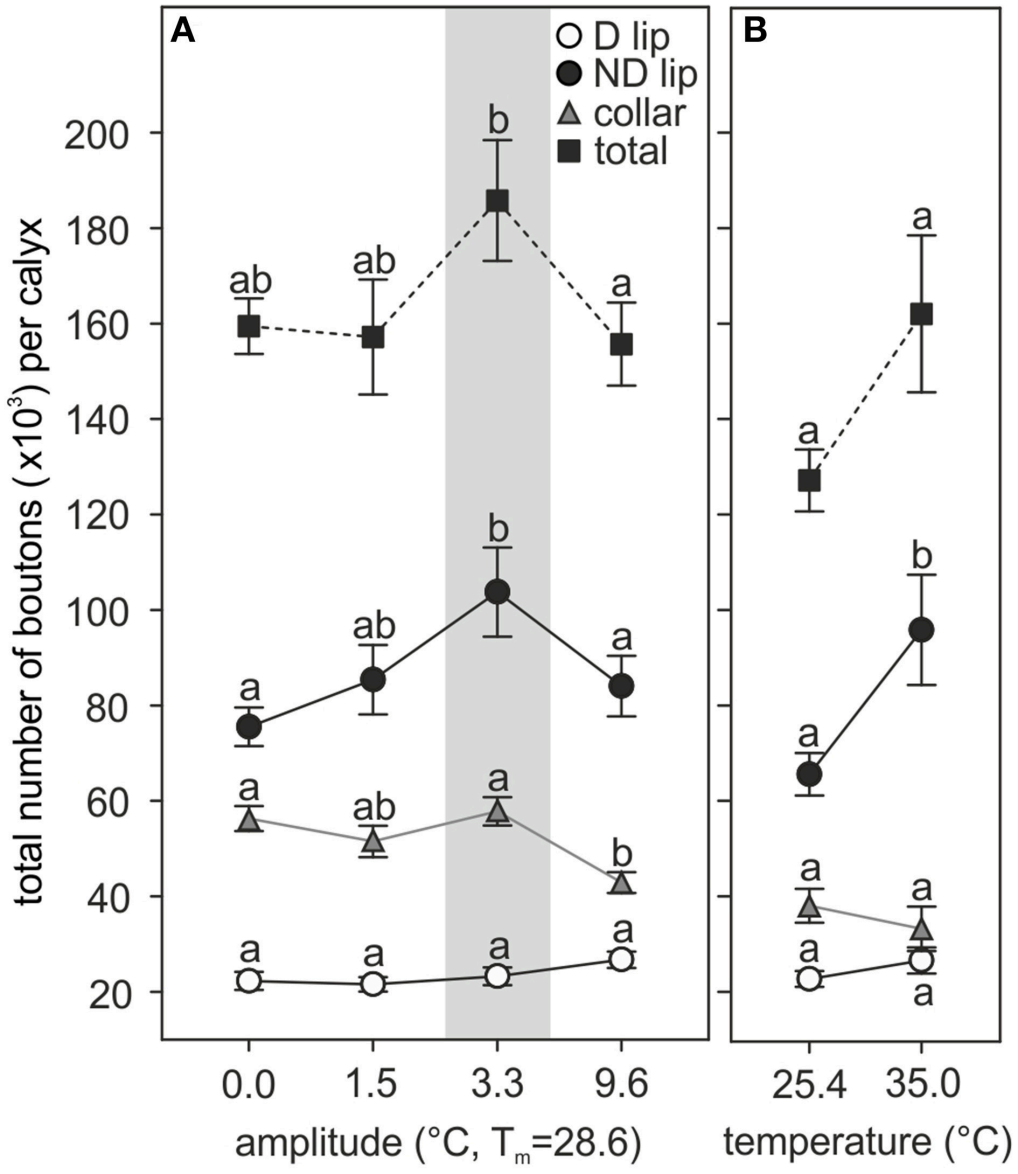
**Pupal thermal experience effects on the number of MB calyx synaptic boutons in the adult brain**. Pupae reared at **(A)** different amplitudes of fluctuating temperature but the same mean temperature or **(B)** different constant temperature regimes. Number of synaptic boutons per calyx in the D lip, ND lip, collar, and the sum of all three regions (total number of boutons per calyx). Gray area shows the temperature regime selected by nurses for brood translocation. Symbols represent the mean value of each group and lines the S.E. Different letters indicate significant differences among amplitudes or between constant temperatures. D and ND lip: *N*_0_ = 13, *N*_1.5_ = 9, *N*_3.3_ = 10, *N*_9.6_ = 11, *N*_25.4_ = 11, *N*_35_ = 10; collar: *N*_0_ = 10, *N*_1.5_ = 8, *N*_3.3_ = 11, *N*_9.6_ = 9, *N*_25.4_ = 11, *N*_35_ = 9; total: *N*_0_ = 10, *N*_1.5_ = 8, *N*_3.3_ = 10, *N*_9.6_ = 9, *N*_25.4_ = 11, *N*_35_ = 9.

Extrapolation of the total number of synaptic boutons per calyx showed significant differences among ants reared at different thermal regimes (Figure 5). As observed for the bouton densities, temperature experienced during the pupal phase had the greatest effect on the ND subregion of the MB calyx olfactory lip in both the fluctuating [*amplitude*: *F*_(3, 37)_ = 4.68, *p* = 0.0072; *covariate size*: *F*_(1, 37)_ = 10.82, *p* = 0.0022; ANCOVA; Figure 5A] and constant temperature series [*temperature*: *F*_(1, 18)_ = 6.50, *p* = 0.02; *covariate size*: *F*_(1, 18)_ = 0.34, *p* = 0.56; ANCOVA; Figure 5B]. The largest total number of ND lip boutons was found in those ants reared under a 3.3°C amplitude (~104,000 boutons per calyx lip), while ants reared under 0.0, 1.5, and 9.6°C amplitude showed on average 73, 82, and 81% of this number. Ants reared at 25.4°C constant temperature had on average 69% of the ND lip boutons when compared with ants reared at 35.0°C (~96,000 boutons per calyx). The total number of boutons in the D lip was not affected by the experienced temperature [fluctuating temperature: *amplitude*: *F*_(3, 37)_ = 1.26, *p* = 0.30; *covariate size*: *F*_(1, 37)_ = 1.27, *p* = 0.27; constant temperature: *temperature*: *F*_(1, 18)_ = 0.71, *p* = 0.41; *covariate size*: *F*_(1, 18)_ = 1.44, *p* = 0.25; ANCOVA]. Assuming that calyx volumes and bouton densities are equal for the medial and the lateral calyx, and for both brain hemispheres, we then estimated a rough total number of lip boutons per brain (extrapolated to all four calyces) of ~508,000 in those ants reared under a 3.3°C amplitude, whereas in those reared at 0.0, 1.5, and 9.6°C amplitude the estimated total numbers averaged ~391,000, ~428,000, and ~443,000 boutons, respectively.

Comparison of the total number of synapsin-IR boutons in the collar revealed that development under high thermal amplitudes (9.6°C) significantly reduced the number of synaptic boutons by 26% compared with the group developed under a 3.3°C thermal amplitude regime [~58,000 boutons per calyx in 3.3°C treatment; *F*_(3, 33)_ = 7.44, *p* = 0.0006; *covariate size*: *F*_(1, 33)_ = 4.44, *p* = 0.043; ANCOVA; Figure 5A]. All other fluctuating temperature regimes did not cause any significant differences in the total number of boutons in the collar. We estimated an average of ~231,000 collar boutons per brain (extrapolated to all four calyces) in ants reared under a 3.3°C amplitude, the thermal program that mimics the nurses' behavior. No differences were found between constant temperatures [total number of synapsin-IR boutons in the collar per calyx, 25.4°C: ~38,000, 35°C: ~33,500, *temperature*: *F*_(1, 17)_ = 0.65, *p* = 0.43; *covariate size*: *F*_(1, 17)_ = 0.01, *p* = 0.94; ANCOVA; Figure 5B].

In total, the MB medial calyx of 2-day old ants reared at a 3.3°C amplitude contained on average ~186,000 synapsin-IR boutons (~743,000 per brain, extrapolated to all four calyces). This number was significantly higher than the total number of boutons found in the medial calyx of ants reared at 0.0 (~159,500 boutons) and 9.6°C (~156,000 boutons) amplitude, but not statistically different from the total number of boutons in ants reared under 1.5°C amplitude regime (~157,000 boutons) [*amplitude*: *F*_(3, 32)_ = 3.68, *p* = 0.022; *covariate size*: *F*_(1, 32)_ = 11.09, *p* = 0.0022; ANCOVA; Figure 5A]. The MB medial calyx of ants reared at 25.4 and 35.0°C constant temperature contained on average ~127,000 and ~162,000 boutons, respectively; these two groups were not statistically different [*temperature*: *F*_(1, 17)_ = 4.08, *p* = 0.06; *covariate size*: *F*_(1, 17)_ = 0.02, *p* = 0.88; ANCOVA; Figure 5B].

## Discussion

We show that daily thermal fluctuations experienced by developing pupae and mediated by circadian rhythmic brood-tending behaviors of nurse ants affect the postembryonic development of the MB calyces in *C. mus* ants. Effects of daily fluctuating temperatures on young adult ants were most evident at the level of density and number of synaptic boutons (MG) of the MB calyx. Most importantly, the effects were temperature- and region-specific. The temperature regimes mimicking the thermal preferences of nurses for brood rearing resulted in the highest densities and numbers of MG compared with all other temperature regimes we applied. Temperature experienced during pupal development particularly affected the ND subregion of the MB calyx olfactory lip, while the D subregion of the lip and MG in the collar were less affected by temperature variations.

### General effects of daily fluctuating temperature regimes on pupal development

The favorable thermal range and the optimal thermal regime for postembryonic development in insects are species-specific. Effects of constant and fluctuating temperature regimes have often been studied by quantifying survival rates (Ratte, 1985). In *C. mus*, brood mortality rate under constant temperature regimes increased when temperature deviated some degrees (to 25.4 or 35.0°C) from the mean temperature (28.6°C) experienced by pupae translocated by nurses. This is in agreement with Roces and Núñez (1989), who compared the effects of increased constant temperatures (20, 25, 30, 35°C) with a 3.3°C amplitude (28.6°C mean temperature) fluctuating temperature regime. In the present study we compared the effects of temperature regimes with different thermal amplitudes around the same mean temperature and found that mortality of *C. mus* pupae was lower under fluctuating temperatures, even when the experienced thermal regime varied between two extreme values (amplitude 9.6°C: 16:8 h / 25.4:35.0°C). This confirms that not only the thermal range is important during development, but also how temperature varies on a daily basis within the favorable thermal range boundaries. We therefore conclude that daily fluctuating temperatures play an important role during postembryonic development of *C. mus* workers.

Changing temperatures were shown to have major effect on postembryonic development in hemimetabolous insects. Crickets, for example, are not restricted in locomotion during the pre-imaginal stages and perform self-determined sun-basking that significantly reduces the developmental time (Remmert, 1985). In social Hymenoptera, the temperature experienced by the immobile brood exclusively depends on the nest environment or on adult worker's thermoregulatory behavior. Although there are marked differences in thermoregulatory preferences among Hymenoptera, thermoregulatory control of brood temperature appears to match a species-specific optimum for brood development. *Apis mellifera* workers maintain the brood nest at a constant temperature of 34.8 ± 0.5°C, while the eusocial wasp, *Vespa vulgaris*, keeps the nest at 30.7 ± 2.5°C. In both cases, precise thermoregulation is maintained only when brood is present, and nest temperatures are more variable when brood is absent (Himmer, 1927). In honeybees, this thermal regime promotes the shortest time for pupal development, the highest emergence rate, and the highest rate of synaptic neuropil development in the MB calyx (Groh et al., 2004). *Solenopsis invicta* fire ants show a preference range of 30–32°C for their brood, and this thermal range matches with the temperature that maximizes colony size under non-limited food conditions (Porter, 1988; Porter and Tschinkel, 1993). *Camponotus rufipes* ants also show daily changes in their thermal preferences, but brood translocation behavior follows a different pattern compared to *C. mus* (Roces and Núñez, 1995; Weidenmüller et al., 2009).

Developmental temperature has a major effect on the adult size in insects (reviewed in Ratte, 1985; Angilletta et al., 2004). However, in holometabolous insects, the size of adults is largely determined by the size of the larva just before entering metamorphosis (i.e., the last larval stage; Nijhout, 2003). Since our experimental individuals were taken once their cocoon was spun, we ruled out possible effects of temperature on adult body size. Conversely, larger workers (which certainly were larger larvae at the time of pupation) required in our experiments more time for completing the pupal development under certain regimes, in comparison with relative small ones subjected to the same thermal regime. As also expected, higher constant rearing temperatures shortened the developmental time in *C. mus*, but no significant differences were observed between the thermal regime with a 3.3°C amplitude and other regimes with larger or smaller amplitudes. In accordance with previous results (Roces and Núñez, 1989), pupae reared under the 3.3°C amplitude regime developed at the same speed as those reared at the corresponding mean constant temperature. This suggests that the thermal regime selected by nurses (compared with a constant thermal regime with the same mean value) should be favorable for other physiological parameters than growth (Roces and Núñez, 1989). Unexpectedly, pupae reared under the highest thermal amplitude (9.6°C) developed significantly slower than pupae reared under low thermal amplitudes (0.0 and 1.5°C), although all regimes averaged the same arithmetic value. This may be due to the long exposition to a relatively low temperature (16 h at 25.4°C) experienced by the pupae under the regime with the largest thermal amplitude, suggesting that the growth rate is not a linear function of temperature. It is argued that the physiological responses to temperature depend on the temperature range considered, as known for other temperature-related responses in several insects, including ants (Bollazzi and Roces, 2011; Falibene and Josens, 2014).

### Daily thermal fluctuations during pupal development affect synaptic organization in the adult mushroom body calyx

The thermal regime imposed by *C. mus* nurses on their brood by translocation led to an increase in MG densities and total numbers in specific regions of the adult MBs. Temperature particularly affected the relatively large ND subregion of the MB calyx olfactory lip, while changes in the D lip and visual collar were less evident. Neuropil volumes were not affected by the temperature experienced during development, except for an increase in the collar volume in ants reared at lower constant temperatures. Similar results were previously found in the honeybee—the MB calyx lip was affected by only slight deviations within and beyond the natural range of constant brood rearing temperature experienced in the hive (Groh et al., 2004, 2006). Interestingly, *C. mus* ants and honeybees perform completely different brood thermoregulation strategies: *C. mus* nurses actively move the brood and expose especially the pupae to precisely controlled thermal fluctuations (Roces and Núñez, 1989), whereas honeybees maintain their brood at precisely controlled high and constant temperatures (Himmer, 1927; Seeley and Heinrich, 1981; Heinrich, 1993). Interestingly, species-specific thermoregulation imposed by nurses, although using different temperature regimes, results in a maximum in the numbers of synaptic boutons in the MB calyx lip both in the honeybee and *C. mus* ant. The olfactory lip region of the MB calyx in young adult honeybees (1-day old) reared under the species-specific thermoregulatory preferences of nurses contains between ~177,000 and ~230,000 boutons (Groh et al., 2012; Muenz et al., 2015), whereas in 2-day old *C. mus* ants this number averaged ~127,000.

Changes in MB-calyx MG could be affected by variations at both pre- (PN) and post- (KC) synaptic sites. During larval-adult metamorphosis the MBs undergo extensive remodeling including major proliferation of KCs, KC dendritic growth, and increasing arborization of PN terminals (Farris et al., 1999, 2004; Schröter and Malun, 2000; Ishii et al., 2005; Fahrbach, 2006; Ganeshina et al., 2006; Groh and Rössler, 2008). In addition to neurogenesis, postembryonic metamorphosis also involves programmed cell death and modification of persistent neurons (Levine et al., 1995; Ganeshina et al., 2000; Fahrbach and Weeks, 2002). All of these processes can be affected by temperature. In general, temperature during development influences both the number (differentiation) and the size (growth) of cells at adulthood (reviewed in Angilletta et al., 2004). In addition, temperature directly affects neuronal function (Montgomery and Macdonald, 1990; Janssen, 1992; Rössler and Bickmeyer, 1993).

Temperature-mediated effects were brain area-specific and even varied within the same modality: affecting mostly MG numbers in the large ND lip while effects on the D lip and the visual collar were less evident. It seems likely that these two subregions of the calyx lip in *C. mus* correspond to the differentially innervated subdomains of the lip in other Hymenoptera (ants: Zube et al., 2008; honeybees: Kirschner et al., 2006; Brill et al., 2013). In the ant *Camponotus floridanus*, for example, the inner part of the lip (which may correspond to the ND lip) is predominantly innervated by PNs of the lateral antennal lobe tract (lALT, nomenclature after Ito et al., 2014), whereas the outer lip (which may correspond to the D lip) is exclusively innervated by the PNs of the medial (m) ALT (Zube et al., 2008). A high MG plasticity in the ND lip may indicate that formation of neuronal circuits receiving convergent input from m- and lALT is more prone to temperature influences during the pupal phase. Neuronal and dendritic remodeling during metamorphosis are cell-specific and precisely timed (Weeks and Levine, 1990), and temperature affects timing of neurite growth during pupal development (Rössler et al., 2000). In the honeybee, the development of PN axonal projections in the MBs develop in the first half of pupal development with slight differences in timing of MB subregions (Schröter and Malun, 2000; Groh and Rössler, 2008). It therefore appears likely that temperature fluctuations cause differential downstream effects in the development of m- and lALT PN axonal projections in the MB calyx lip in *C. mus*. Differential development of the lip subregions is also expected to be associated with changes in processing of odor information. In the honeybee, the two ALTs transfer different features of similar odor information: the lALT codes for generalized odor properties while the mALT shows higher odor specificity (Brill et al., 2013, 2015; Rössler and Brill, 2013). Lesion studies also indicate that the two ALTs may also carry different behavioral attributes (Carcaud et al., 2016).

### Effects of pupal thermal experience on adult behavior

Temperature experienced during pupal development affects adult behavior in social Hymenoptera. Weidenmüller et al. (2009) showed that *C. rufipes* worker ants reared at different constant temperatures only during their pupal phase have different response thresholds to increasing temperatures and different temperature preferences for brood translocation as adults. This behavior was observed as late as 33 days after adult eclosion. Thus, pupal thermal experience may cause long lasting behavioral changes in ants. In honeybees, adult behavioral performance such as waggle-dance behavior, learning, and short-term memory formation are impaired in adults that experienced suboptimal temperatures during pupal development (Tautz et al., 2003; Jones et al., 2005; Becher et al., 2009). Interestingly, enhanced behavioral performance of workers reared under optimal thermal regimes (i.e., the temperature normally maintained in brood cells) correlated with the highest number of MG in the adult brain (Groh et al., 2004). Differences in the initial numbers of MG in the MB calyx may have important consequences for later stages of adulthood, for example during sensory-exposure induced synaptic pruning (Stieb et al., 2010, 2012; Scholl et al., 2014; Falibene et al., 2015; Yilmaz et al., 2016) or the MG formation during associative learning and long-term memory formation (Hourcade et al., 2010; Falibene et al., 2015). In more general terms, a high initial number of MG synaptic connections in young adults may provide larger computational capacities during subsequent adulthood (Chittka and Niven, 2009). As a consequence of natural variations in brood rearing temperatures, differences in pupal thermal experiences may as well promote interindividual variability in behaviors influencing division of labor (Jeanson and Weidenmüller, 2014). This could be particularly relevant for ant colonies with brood distributed in several nest chambers and subjected to different daily and seasonal thermoperiodicities, such as those of several *Camponotus* species, in which nurse workers may not always find their preferred temperatures for the brood. We conclude that brain-region and modality specific effects of developmental temperature regimes on synaptic development in the ant brain represent an important source of interindividual variability in behavior.

## Author contributions

AF, FR, CG, and WR designed the experiments. AF performed all experiments, evaluated, and analyzed the data, and drafted the MS. WR, FR, and CG revised the work critically for important intellectual content and edited the manuscript. All authors agreed on the final version of the MS.

### Conflict of interest statement

The authors declare that the research was conducted in the absence of any commercial or financial relationships that could be construed as a potential conflict of interest.
